# Biological and Mechanical Properties of Platelet-Rich Fibrin Membranes after Thermal Manipulation and Preparation in a Single-Syringe Closed System

**DOI:** 10.3390/ijms19113433

**Published:** 2018-11-01

**Authors:** Dorottya Kardos, István Hornyák, Melinda Simon, Adél Hinsenkamp, Bence Marschall, Róbert Várdai, Alfréd Kállay-Menyhárd, Balázs Pinke, László Mészáros, Olga Kuten, Stefan Nehrer, Zsombor Lacza

**Affiliations:** 1 Institute of Clinical Experimental Research, Semmelweis University, 1094 Budapest, Hungary; istvan.hornyak@orthosera.com (I.H.); melinda.simon@orthosera.com (M.S.); adel.hinsenkamp@orthosera.com (A.H.); marschall.bence@gmail.com (B.M.); zsombor.lacza@orthosera.com (Z.L.); 2 Orthosera GmbH, 3500 Krems an der Donau, Austria; olga.kuten@orthosera.com; 3 Department of Physical Chemistry and Materials Science, Budapest University of Technology and Economics, 1111 Budapest, Hungary; vardai.robert@mail.bme.hu (R.V.); AMenyhard@mail.bme.hu (A.K.-M.); 4 Institute of Materials and Environmental Chemistry, Research Centre for Natural Sciences, Hungarian Academy of Sciences,1117 Budapest, Hungary; 5 Department of Polymer Engineering, Faculty of Mechanical Engineering, Budapest University of Technology and Economics, 1111 Budapest, Hungary; pinke@pt.bme.hu (B.P.); meszaros@pt.bme.hu (L.M.); 6 Center for Regenerative Medicine, Danube University, 3500 Krems-an-der-Donau, Austria; stefan.nehrer@donau-uni.ac.at; 7 Institute of Sport and Health Sciences, University of Physical Education, 1123 Budapest, Hungary

**Keywords:** biopolymer, platelet-rich fibrin, scaffold, biodegradation, tissue regeneration, medical device

## Abstract

Platelet-rich fibrin (PRF) membrane is a three-dimensional biodegradable biopolymer, which consists of platelet derived growth factors enhancing cell adhesion and proliferation. It is widely used in soft and hard tissue regeneration, however, there are unresolved problems with its clinical application. Its preparation needs open handling of the membranes, it degrades easily, and it has a low tensile strength which does not hold a suture blocking wider clinical applications of PRF. Our aim was to produce a sterile, suturable, reproducible PRF membrane suitable for surgical intervention. We compared the biological and mechanical properties of PRF membranes created by the classical glass-tube and those that were created in a single-syringe closed system (hypACT Inject), which allowed aseptic preparation. HypACT Inject device produces a PRF membrane with better handling characteristics without compromising biological properties. Freeze-thawing resulted in significantly higher tensile strength and higher cell adhesion at a lower degradation rate of the membranes. Mesenchymal stem cells seeded onto PRF membranes readily proliferated on the surface of fresh, but even better on freeze/thawed or freeze-dried membranes. These data show that PRF membranes can be made sterile, more uniform and significantly stronger which makes it possible to use them as suturable surgical membranes.

## 1. Introduction

Hard and soft tissue regeneration is mediated by a wide range of signaling events, which are regulated by numerous cytokines. Altough the origin of these molecules is not well-known, platelets have been found to be a key autologous source of growth factors [1,2]. Upon activation platelets secrete active proteins including platelet-derived growth factor (PDGF), transforming growth factor (TGF β), and insulin-like growth factor (IGF-I) [3]. Autologous platelet concentrates are widely used as bioactive surgical additives to decrease inflammation and increase the speed of healing processes [4]. Several different techniques have been developed to produce platelet concentrates from blood [5,6].

The first generation platelet concentrate, platelet-rich plasma (PRP) was introduced by Marx et al. in 1998. PRP is used with bone grafts to repair osseous defects in dentistry, in maxillofacial/ENT-surgery, and in orthopedics [7,8,9]. It is also used to accelerate soft tissue regeneration, like the facilitation of gingival and dermal wound healing [10,11] and PRP gains traction as an injectable therapy for osteoarthritis. The disadvantages of using PRP are the lack of uniformity in PRP preparation methods and the use of bovine thrombin for activation [12]. Platelet rich fibrin (PRF) can be described as a second generation autologous platelet concentrate because it does not require any biochemical additives like anticoagulants or bovine thrombin. The preparation of PRF by immediate centrifugation of venous blood collected in glass tubes was first described by Choukroun et al. in 2000 [13]. Soluble fibrinogen found in the plasma transforms to fibrin, which polymerizes to a three-dimensional structure [14]. During centrifugation, the activated platelets and some leukocytes are entrapped in the fibrin matrix. Consequently, a storage pool of growth factors is formed from platelets upon activation. These molecules act as cell attractants and they increase the rate of the proliferation of adhered cells [15]. Serum squeezed out from the PRF clot, called hyperacute serum, has higher cell proliferative effect on bone marrow mesenchymal stem cells (MSCs), osteoblasts and osteoarthritic chondrocyte cells than PRP [16,17]. After removing the hyperacute serum fraction, the remaining PRF membrane is a three-dimensional, biocompatible, biodegradable scaffold, which can slowly and sustainably release bioactive molecules, which facilitate cell adhesion and proliferation [18,19]. PRF membrane can provide an appropriate cell-niche for most cell types, because the outer layer allows contact of cells and ECM molecules through the surface molecules of the membrane. Thus the surface of PRF membrane supports cellular functions such as adhesion and migration [20,21].

The application of PRF membrane in oral and maxillofacial surgery is widespread, however, the right application of PRF and the outcome of the surgery also depends on the preparation method of the membrane, and also from the ability of surgeons, and from the complexity of the operation [22,23,24,25,26]. The membrane is capable of increasing the adhesion and proliferation capacity of gingival fibroblasts, thus PRF membrane is suitable for periodontal regeneration [27,28,29]. Furthermore PRF increases new bone formation and has a positive effect on early bone healing [30,31]. PRF is also suitable to facilitate meniscal repair by promoting meniscocytes proliferation [32], and dermal fibroblast migration during the remodeling of the skin [33,34,35]. Li Q et al. [36] compared the effects of fresh and lyophilized PRF on dental follicle, periodontal ligament and alveolar bone cells. They concluded that lyophilized PRF has the benefit of improved storage capacity of growth factors and possess an increased osteogenic potential compared to fresh PRF. Freeze-drying does not influence the stability of biofactors, the structure of fibrinogen and the clinical applicability of PRF [37].

Despite the very clear regenerative advantages of the PRF membrane, extending its use towards other surgical fields has several roadblocks. First, the preparation of PRF requires open handling of the fibrin clot which breaks the sterile barrier and can only be performed in a sterile cabinet, effectively limiting its use to the oral cavity where surgery is not aseptic anyway. Second, the structure of the membrane is not suitable for wound closing, because it has low and variable tensile strength so the material does not hold a suture [38]. Furthermore the degradation time of PRF cannot be influenced in a natural way without causing changes in the structure of the membrane itself [39]. Therefore, the aim of the present study was to produce a sterile, suturable, homogenous PRF membrane suitable for surgical intervention. We have also investigated how simple thermal preservation methods such as freezing and freeze/drying affect the porperties of PRF.

## 2. Materials and Methods

### 2.1. Platelet-Rich Fibrin Membrane Preparation

**Figure 1:**
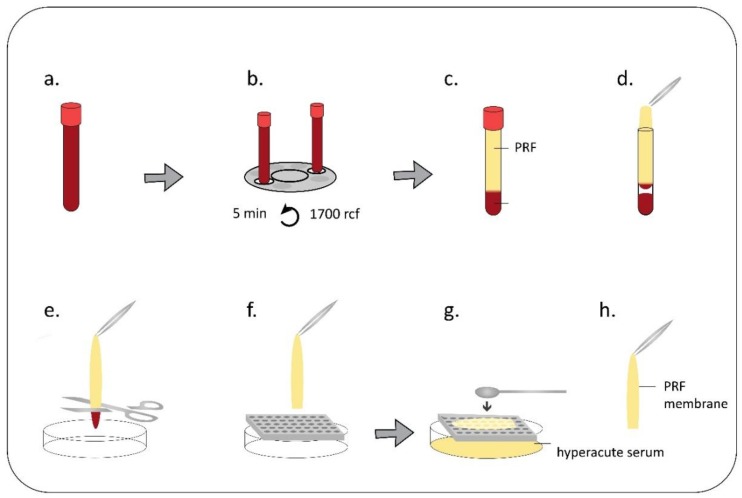
PRF membrane preparation by glass blood drawing tube. 8 mL venous blood was taken by venipuncture from healthy volunteers into glass tube (**a**) and it was immediately centrifuged (1700 rcf, 5 min) (**b**). After centrifugation two layers were observed in the tubes. The top layer was the platelet-rich fibrin clot, the base layer was the red blood cells (**c**). The PRF was removed by sterile forceps in a laminar flow tissue culture hood (**d**), red blood cells at the bottom of the fibrin clot were cut away (**e**) and the clot was placed onto a sterile grid (**f**). The hyperacute serum was squeezed out from the PRF clot by sterile spatule (**g**). The remaining film was the PRF membrane (**h**). (Figure 1, Figure S1A).

**Figure 2:**
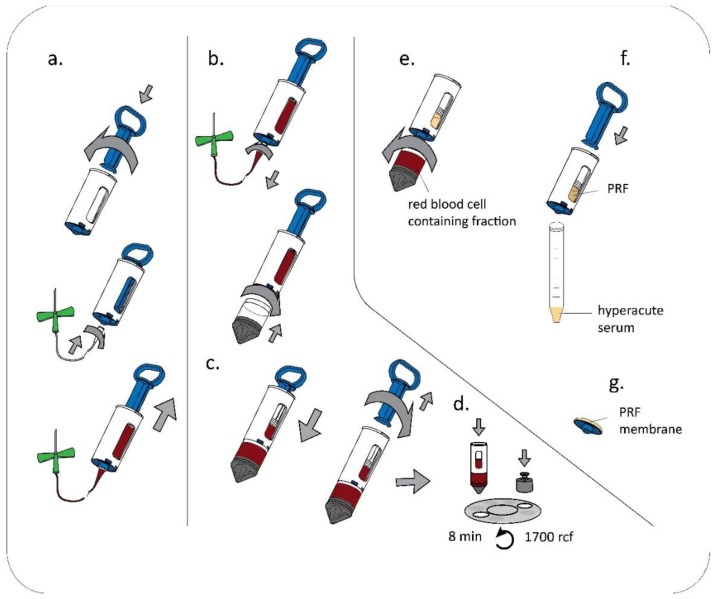
PRF membrane preparation by a single-syringe closed system (hypACT Inject Auto). 18 mL venous blood was taken by venipuncture from healthy volunteers into the hypACT Inject syringe (**a**). The waste container was attached to the syringe (**b**) and the whole blood was pushed down by the plunger manually until the waste container was filled with blood (**c**). The syringe was immediately centrifuged (1700 rcf, 8 min) (**d**). After centrifugation, whole blood became separated to red blood cells and supernatant, the waste container contains the red blood cells, the syringe contains the supernatant. PRF is formed from the supernatant due to natural clotting. After this, the waste container was removed (**e**) and hyperacute serum was pressed out from the syringe by re-attaching and pressing the plunger (**f**). The lower plastic cap, which holds the filter was removed in a sterile laminar flow tissue culture hood and the PRF membrane was pulled off by sterile forceps from the inner side of the plastic cap (**g**). (Figure 2, Figure S1B).

Blood samples were obtained from healthy donors aged 24–45 years under IRB approval (IRB approval number 33106-1/2016/EKU, 12.07.2016.). The classical preparation method of PRF is shown in Figure 1, while preparation by the single-syringe closed system in Figure 2. Actual pictures of the preparations and the devices are provided in Supplementary Figure S1.

### 2.2. Sample Preparation

Fresh, frozen, and freeze-dried PRF membranes were used during the experiments. Fresh membranes were used immediately after isolation. Frozen membranes were frozen at −20 °C, overnight and thawed at +4 °C. Freeze-dried membranes were frozen at −80 °C for 30 min and freeze-dried (−54 °C, 12 Pa) overnight. Before the experiment freeze-dried PRF membranes were moistened with phosphate buffered saline without Mg^2+^, Ca^2+^.

## 3. Evaluation of Mechanical and Structural Properties

### 3.1. Tensile Strength Measurements

Tensile test of fresh, frozen and freeze-dried PRF membrane from either glass blood drawing tube (GT) or hypACT Inject (HI) was assessed using universal testing machine (Instron 5566). The samples (diameter: 10 mm, length: 25 mm) were fixed with clamps at both ends (using slip-proof rubber sheets to prevent slipping), the initial distance between clamp faces was set to 5 mm for all the samples tested. Tensile loading was applied at a cross head speed of 10 mm/min; the maximum load at specimen failure was recorded and tensile strength was calculated using the following equation: $S=\frac{F}{A}$, F is maximum force (N) and A is unit area (m^2^). Stress-strain curve was recorded with Instron Bluehill 3 software (Grove City, PA, USA) simultaneously.

We tried to suture the fresh GT and HI PRF membranes into hyaline cartilage by Nylon (Ethilon) Black Monofilament, 6-0, single-armed P1 needle. (Ethicon Inc., Somerville, NJ, USA). Hyaline cartilage defects were created manually in knee joint tissues, which were excised surgically during a routine knee replacement operation (Department of Orthopedics, Faculty of Medicine, Semmelweis University, Budapest, IRB approval number 33106-1/2016/EKU, 12.07.2016.).

### 3.2. Scanning Electron Microscopic Observation

The surface microstructure of fresh, frozen and freeze-dried GT and HI PRF membrane were examined by a scanning electron microscope (SEM) (JEOL JSM-6380LA). Before microscopic observation the membranes were fixed by 2.5% glutaraldehyde for 20 min. Dehydration was performed with increasing concentrations of ethanol (50%, 70%, 80%, 90%, 100%) for 5 min each. After dehydration, samples were treated with 0.5 mL of 100% hexamethyldisilazane (HMDS) for 5 min and they were left in the safety cabinet overnight to allow excess HMDS to evaporate. After drying, the samples were sputter-coated with gold (JEOL JFC-1200 Fine Coater, 12 mA, 20 s) and examined under SEM. The middle region of the surface and cross section of each membranes were scanned using 500× and 5000× magnification.

## 4. Evaluation of Biological Properties

### 4.1. Live/Dead Staining

Cellular viability of blood cells embedded into the PRF membrane was measured with the Live/Dead cell viability assay. Membranes were washed 3 times with PBS, and stained in PBS contaning 1 μM Calcein-AM (Invitrogen, Carlsbad, CA, USA) and 5 μg/mL ethidium homodimer (Invitrogen, Carlsbad, CA, USA) for 30 min. The samples were washed three times with PBS again. Confocal Z-stack images were taken using 20× magnification with Nikon A1R confocal microscope (Nikon-KOKI Imaging Center, Budapest, Hungary). The Z-stack images were visualized as maximal intensity projection using Fiji-ImageJ software.

### 4.2. Mesenchymal Stem Cell Culture on PRF Membranes

Bone marrow derived mesenchymal stem cells (MSCs, ATCC, Manassas, VA, USA) were cultured using T-75 culture flasks in an incubator at 37 °C, 5% CO_2_ at 95% humidity. Cells were maintained in Dulbecco’s modified Eagle’s medium (DMEM) containing 4.5 g/L glucose, pyruvate, GlutaMAX^TM^ (Gibco, Paisley, Scotland) supplemented with 10% fetal calf serum (FCS) (Gibco, Paisley, Scotland), 2 μL/mL Primocin (Invitrogen, Carlsbad, CA, USA), and 0.75 ng/mL basic fibroblast growth factor (bFGF) (Sigma-Aldrich, St. Louis, MO, USA). Cell culture medium was refreshed twice a week. MSCs were seeded onto fresh, frozen, and freeze-dried PRF membranes in 24 well low attachment plates at a density of 35,000 cells/membrane. Cell viability on 1, 7, and 14 days was determined using Cell Proliferation Kit II (XTT; Roche, Mannheim, Germany) according to the manufacturer’s instructions. Absorbance was measured after four hours’ incubation in the staining solution using a PowerWave XS microplate spectrophotometer (BioTek, Winooski, VT, USA) at 480 nm with a reference wavelength at 650 nm. The wet weight of the PRF membranes were measured on 1, 3, 6, 8, 10, and 13 days during cell culture period using a digital analytical balance.

### 4.3. Gingival Fibroblast Culture

Gingival fibroblasts (ATCC, Manassas, VA, USA) were cultured using T-75 culture flasks in an incubator at 37 °C, 5% CO_2_ at 95% humidity. Cells were maintanied in Dulbecco’s modified Eagle’s medium Ham’s F-12 1:1 Mix (DMEM/F-12) containing 12 mM Hepes and L-Glutamin (Lonza, Walkersvillw, MD, USA) supplemented with 10% FCS, 50 μg/mL L-ascorbic acid-2-phosphate (Sigma-Aldrich, St. Louis, MO, USA), 2 μL/mL Primocin (Invitrogen, Carlsbad, CA, USA), and 0.75 ng/mL bFGF. Cell culture medium was refreshed twice a week. Gingival fibroblasts were seeded onto fresh, frozen and freeze-dried PRF membranes in 24 well low attachment plates at a density of 35,000 cells/membrane. Cell viability on 1, 3, and 7 days in culture was determined using Cell Proliferation Kit II as described above. Cell culture supernatant of gingival fibroblasts was collected on days 3, 5, and 7. Collagen type I in the supernatant was determined by the Human Pro-Collagen I alpha 1 ELISA KIT (Abcam, Cambridge, UK).

### 4.4. Plasmin Activity Measurement

For the plasmin activation assay, 157.5 μL dimethyl sulfoxide (DMSO) (Sigma-Aldrich, St. Louis, MO, USA) was added to 5 mg *N*-(*p*-Tosyl)-Gly-Pro-Lys 4-nitroanilide acetate salt (Sigma-Aldrich, St. Louis, MO, USA) to get a 50 mM substrate solution. 50 mg PRF membrane was mixed with 97 μL of assay buffer (0.1 M NaPO_4_, pH 7.8, made from Na_2_HPO_4_ and NaH_2_PO_4_; Sigma-Aldrich, St. Louis, MO, USA) and 2 μL of substrate solution, and the absorption at a wavelength of 410 nm was recorded kinetically with a PowerWave XS microplate spectrophotometer. In case of frozen PRF membrane samples, six different samples were produced, with combining the freezing (−20 °C or −80 °C) and thawing (+4 °C, +25 °C, +37 °C) temperatures. Plasma samples were used as negative control and serum samples as positive control.

## 5. Statistical Analysis

One-way analysis of variance (ANOVA) was performed with Tukey’s post hoc test to compare means of the groups. Significance level was *p* < 0.05. Prism software (Irvine, CA, USA) was used for statistical analysis. Data are presented as mean ± SEM.

## 6. Results

### 6.1. Evaluation of Mechanical and Structural Properties

**Figure 3:**
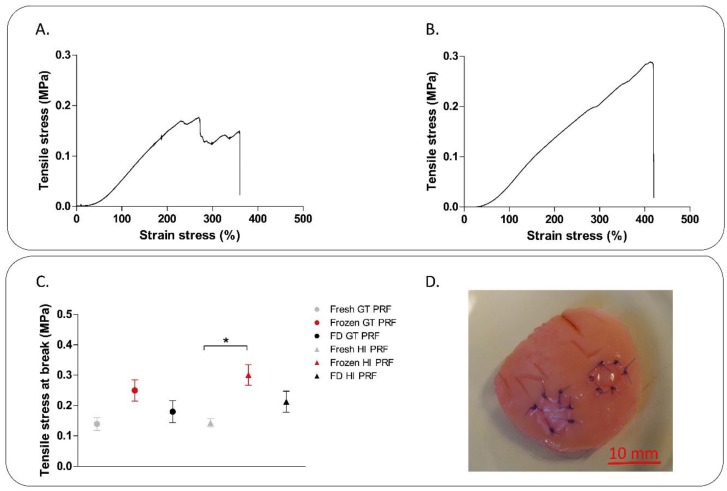
Tensile strength measurement of PRF membrane. (**A**,**B**) show the typical tensile curves of PRF membrane produced by glass blood drawing tube (**A**) and hypACT Inject (**B**) and the tensile strength of the membranes (**C**), where the maximum load at specimen failure was recorded and tensile strength was calculated. (**D**) shows the fresh HI PRF membrane sutured into hyaline cartilage defects.

First the mechanical properties of the PRF membranes were set out to analyze. As the typical stress strain curve of GT (Figure 3A) and HI membranes (Figure 3B) shows, both of GT and HI PRF membrane could be stretched three to four times their original length. The fibers of GT PRF membrane were torn apart at different time points, while in case of HI PRF membrane the whole material torn apart along a line in the center of the membrane at once. HI PRF membranes had the same resistance against tensile stress at each point of the material. There was some variation in tensile strength of fresh, frozen and freeze-dried samples, however significant difference was found only between frozen and fresh HI PRF membranes (Figure 3C). Only HI PRF membrane were suturable, when we tried to suture it into hyaline cartilage defects (Figure 3D).

**Figure 4:**
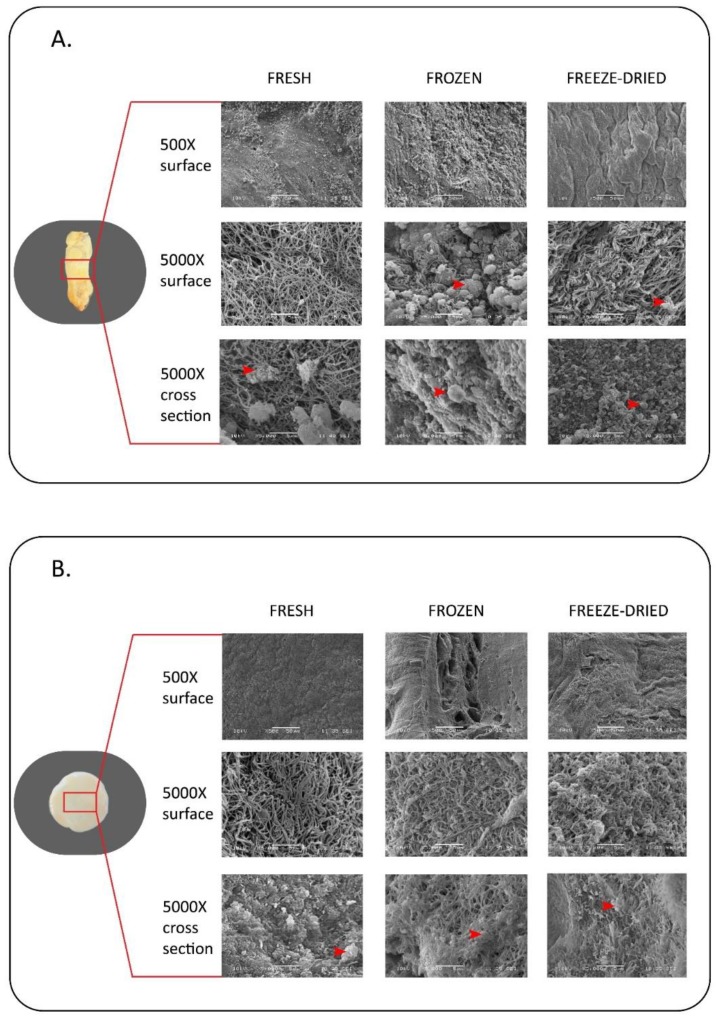
Scanning electron microscope imaging of PRF membranes. The surface microstructure of fresh, frozen and freeze-dried PRF created by the classical glass tube method (GT) is shown in (**A**) and PRF created by the single-syringe closed system (HI) on (**B**). The middle portion of the surface of each membrane was scanned using 500× and 5000× magnifications. The cross-sections of the samples were monitored using 5000× magnification. Red arrows show the platelets on the surface and on the inside of the membranes.

Structural analysis was performed by scanning electron microscopy. We investigated the surface and the fibers of the fibrin membranes, furthermore we examined the location of platelets on the surface and inside of the membranes. We found that frozen and freeze-dried samples have rugged surface, while the structure of these samples are more compact with smaller pores between the fibers, compared to fresh membranes. Platelets can be observed both on the surface and inside of the GT PRF membranes (Figure 4A), however, in the case of HI PRF (Figure 4B), platelets are located mainly inside the membranes.

### 6.2. Evaluation of Biological Properties

**Figure 5:**
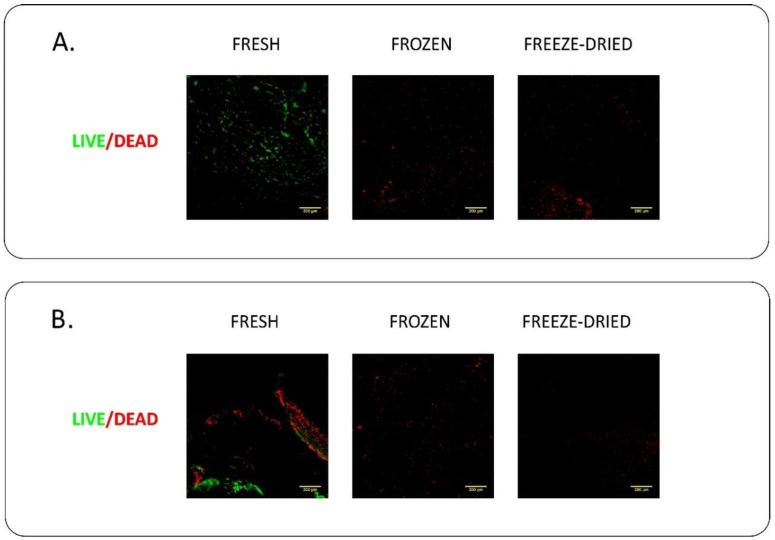
Live/dead cell imaging of the membranes by confocal microscope. Cellular viability of leukocytes embedded into the GT (**A**) and HI (**B**) PRF. Living cells were only observed in fresh samples.

We verified whether the leukocytes in fresh, frozen and freeze dried PRF membranes are alive or dead. Living cells were observed only in fresh PRF membranes. While freezing/thawing and freeze-drying induced the disruption of leukocytes embedded in the PRF membrane. As shown in Figure 5, only disrupted dead cells were present in frozen and freeze-dried PRF membranes.

**Figure 6:**
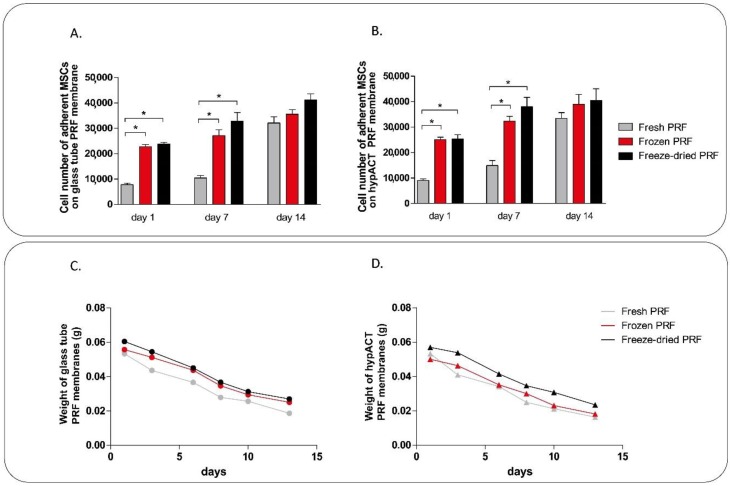
The adhesion and proliferation capacities of mesenchymal stem cells cultured on PRF membranes (**A**,**B**) and weight loss of the membranes (**C**,**D**) during the culture period. Mesenchymal stem cells were seeded onto fresh, frozen, and freeze-dried GT and HI PRF membranes in 24 well low attachment plates. Significant differences (*p* < 0.05) were marked by *.

For its application in tissue engineering, cell adhesion and proliferation capacity are important properties of PRF membranes. Since mesenchymal stem cells are one of the most relevant cell type in tissue regeneration, we put bone marrow derived MSCs onto the membranes to investigate cell adhesion rate in one day and cell proliferation capacity within two weeks. Both the MSC adhesion on day 1 and the proliferation on day 7 were significantly higher in frozen GT and freeze-dried and HI PRF membranes, than in case of fresh samples (Figure 6). There was no significant difference between the samples on day 14, by which time all preparations had comparable cell densities, probably approaching confluence. There was no difference in MSC adhesion and proliferation between GT and HI PRF membranes (Figure 6). The membranes lost their weight during the culture period because both cultured cells and the innate plasmin activity could degrade fibrin. Thus, we measured the weight loss of the membranes during the culture period. Despite the different cell adhesion and proliferation capacities, weight loss of PRF membranes were not significantly different (Figure 6).

**Figure 7:**
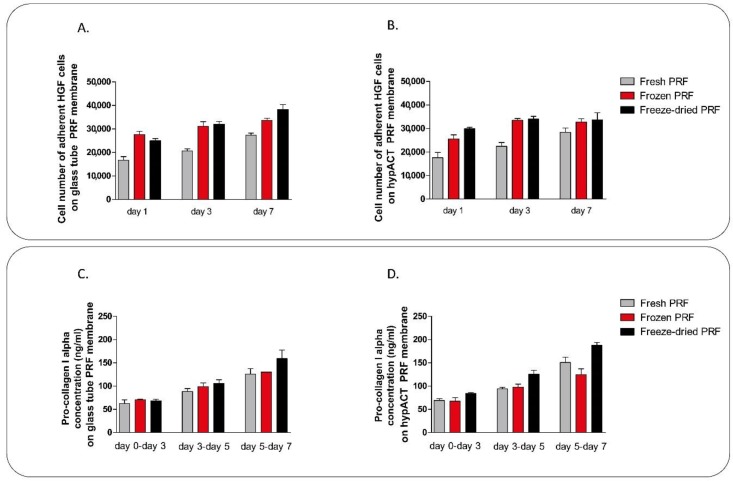
Adhesion and proliferation capacities of human gingival fibroblasts cultured on GT (**A**) and HI (**B**) PRF membranes. Pro-collagen I alpha secretion of the fibroblasts on GT (**C**) and HI (**D**) PRF during the culture period. Although a trend was observed, this does not reach the level of significance between fresh and frozen or freeze/dried samples.

The most widespread clinical application area of PRF membranes is in dentistry as adjuvants to gingival repair. For this reason we cultured human gingival fibroblasts on it, where we examined the adhesion and proliferation rate of cells. Unlike the differences observed in MSCs adhesion capacity, we found no significant differences in the adhesion and proliferation rate in case of gingival cells (Figure 7). However, the cell number of adhered cells within one day was lower on fresh PRF membrane than on frozen or freeze-dried ones (Figure 7). We also measured the pro-collagen I alpha secretion of cells in the cell culture supernatant. We found no significant differences in pro-collagen I alpha production of HGF cells cultured on fresh, frozen and freeze-dried GT and HI PRF. However, comparing pro-collagen production considering the different number of adherent cells on fresh, frozen, and freeze-dried membranes, we found that HGF cells, cultured on frozen PRF membrane could produce lower amount of collagen, than cells on fresh and freeze-dried membranes (Figure 7C,D).

**Figure 8:**
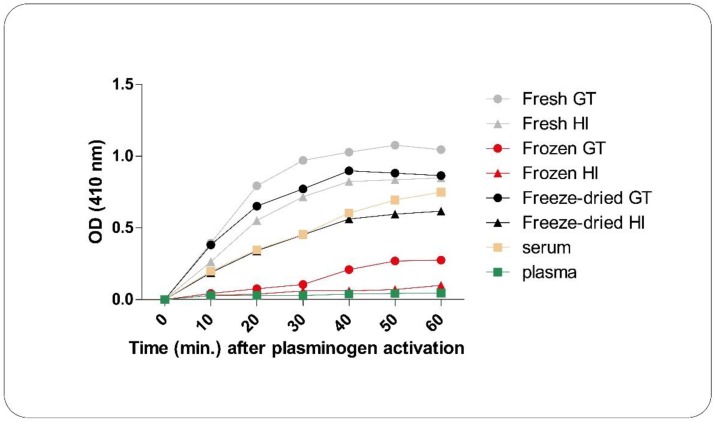
Plasmin activity of fresh, frozen and freeze-dried GT and HI PRF membranes. We used plasma samples as negative control and serum samples as positive control.

Higher plasmin enzyme activity leads to faster degradation, which is a crucial point in tissue repair. The frozen and thawed PRF membrane has significantly lower plasmin activity than that of either fresh or freeze-dried ones. Freeze-drying in itself had no effect on plasmin activity (Figure 8). We investigated whether the different freezing and thawing temperatures can influence enzyme activity. Freezing at −20 °C and thawing at +4 °C is the optimum temperature to decrease the plasmin activity, however the differences in enzyme activity between the various freezing and thawing temperatures were modest and did not reach the level of significance (Supplementary Figure S2).

## 7. Discussion

Structural and tensile strength properties of medical biomaterials are particularly important because of the clinical and surgical usability. On the basis of the mechanical and structural evaluation HI PRF membrane has the same characteristics as GT PRF membrane. However, on the basis of the tensile strength measurements HI PRF membrane is more homogeneous compared to GT PRF. Among the samples only frozen HI PRF has significantly higher tensile strength compared to the fresh HI membrane. Thus, frozen HI PRF would be the most preferable to use as scaffold for tissue repair when high tensile strength is an issue, such as suturing. The tensile strength and stretching properties of our GT PRF membrane is very similar to PRF membranes used in earlier studies, while HI PRF membrane has better mechanical properties in most cases than membranes produced by other techniques [40,41]. SEM imaging showed the structural differences between fresh, frozen, and freeze-dried samples. Frozen and freeze-dried PRF have a more compact structure and rugged surface compared to the fresh one, which may cause the difference in tensile strength properties. The distribution of platelets is more consistent in HI PRF membrane than in GT PRF, which can lead to proportional growth factor release from each areas of the membrane. Unlike our results, Li et al. found that, comparing the structural properties of fresh and lyophilized PRF membranes, fresh membrane has more compact structure with larger pores between the fibers. The explanation of this difference is, that Li et al. monitored dry lyophilized membranes, while our freeze-dried membranes were moistened with PBS before SEM imaging [36].

The growth factors released from the platelets act as cell attractants and cell proliferation agents. If the cell adhesion and proliferation rate on the membrane is slower than the degradation of the scaffold, complete regeneration cannot occur. Comparing the cell adhesion and proliferation abilities of PRF membranes we cultured human mesenchymal stem cells and human gingival fibroblasts on the different membranes. As expected, we found no differences between GT and HI PRF membranes in this regard since their composition is essentially the same. Lower number of cells could adhere onto fresh PRF membranes than onto frozen and freeze-dried ones, however, the difference was significant only in case of MSCs. This result was also confirmed by previous studies [36]. The live/dead cell staining of the membrane without cultured cells on it showed that living cells have been found in fresh membranes only, while frozen and freeze-dried membranes contained only dead cells. In case of human gingival fibroblast culture, we found higher pro-collagen I alpha production on days 3 and 5 in case of fresh membranes, but we found no significant differences between fresh, frozen and freeze-dried samples.

The degradation time of biodegradable materials, such as PRF membrane is also not negligible in surgical interventions. If the material degrades more rapidly than new tissue can be formed in its place, it leads to insufficient tissue regeneration. PRF clot degraded both by the plasmin enzyme that is embedded in the membrane and also by the adhered cells. On the basis of our results plasmin enzyme has significantly lower activity in frozen/thawed membranes, than in fresh and freeze-dried membranes, which causes lower degradation rate. It is probable that thawing but not freezing damages the structure of the enzyme, since freeze-drying did not show the same effect as freeze/thawing. Freezing of the membranes at −20 °C and thawing at +4 °C may help to solve the problem of rapid degradation. This allows the tuning of the membrane for a desired clinical property (e.g., fast or slow degradation) by simple physical means.

We conclude that the hypACT Inject Auto device provides an opportunity for preparing a sterile, reproducible PRF membrane with better mechanical properties and with the same biological properties as in case of PRF membranes produced by glass blood drawing tube. Freeze/thawing and freeze-drying processes cause changes both in the structural, mechanical, and biological properties of PRF membranes. Freeze-thawing results in significantly higher tensile strength, significantly higher cell adhesion and lower degradation rate. Thus, it is an advantageous preparation method to help in the application of an autologous biocompatible membrane for tissue regeneration. In the following experimental steps this membrane can be useful to start clinical studies to investigate the effect of frozen hypACT PRF membrane as gingival graft after bone replacing by albumin-coated bone granules in maxillofacial surgery [42,43,44,45], the material is also a promising candidate for clininal studies in orthopedics, namely in cartilage regeneration.

## Supplementary Materials

The following are available online, Figure S1: PRF membrane preparation by glass blood drawing tube (A) and by a medical device, called hypACT Inject (B), Figure S2: Plasmin activity of frozen GT (A) and HI (B) PRF membranes.
