# Supplementary material for: Biological and Mechanical Properties of Platelet-Rich Fibrin Membranes after Thermal Manipulation and Preparation in a Single-Syringe Closed System
Source: Int J Mol Sci. 2018 Nov 1;19(11):3433. doi: 10.3390/ijms19113433 (PMC6274993; doi:10.3390/ijms19113433)
Supplement: Supplementary file 1 [file ijms-19-03433-s001.pdf]

Supplementary Material

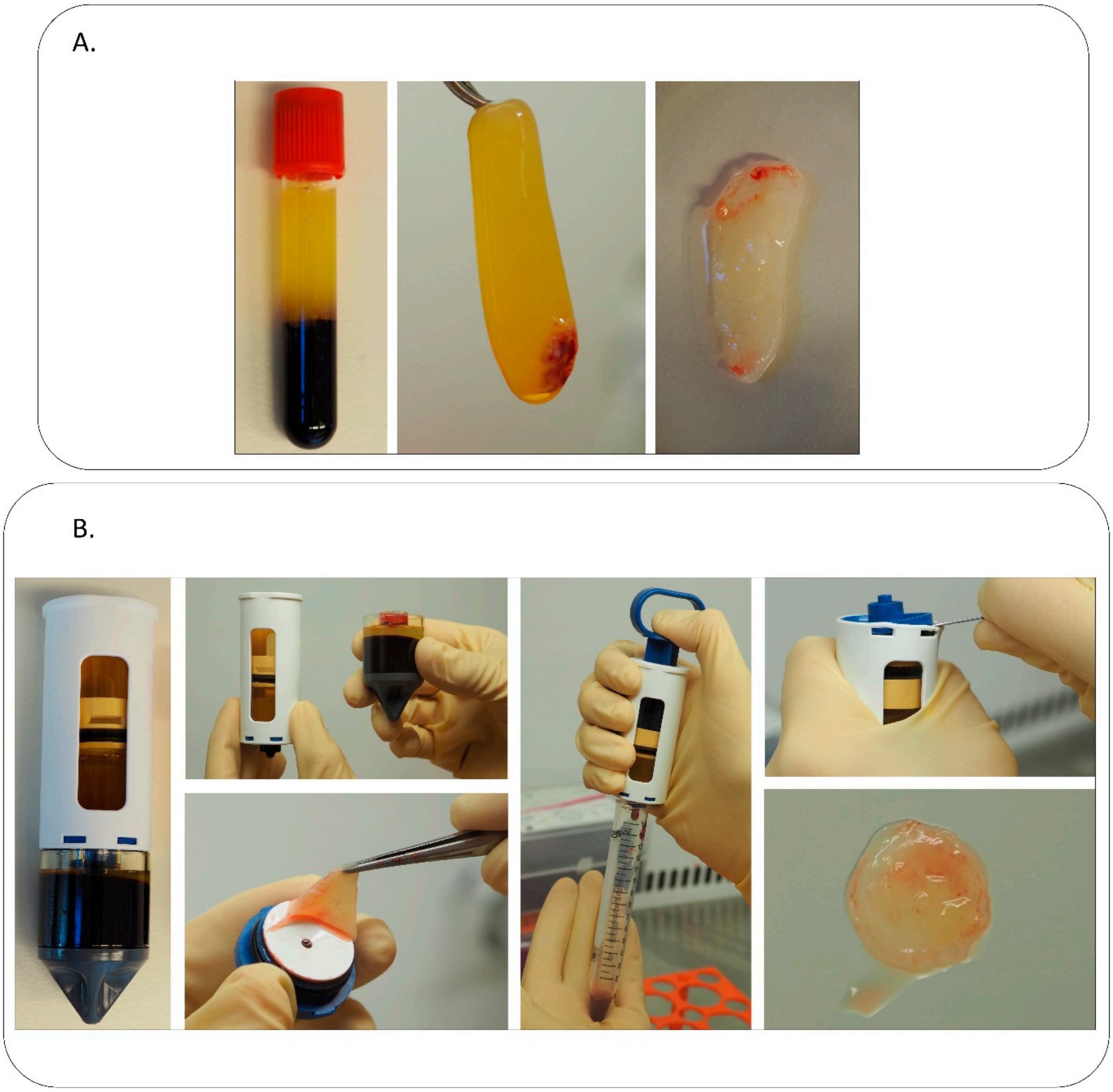

Figure S1. PRF membrane preparation by glass blood drawing tube (A.) and by a medical device, called hypACT Inject (B.).

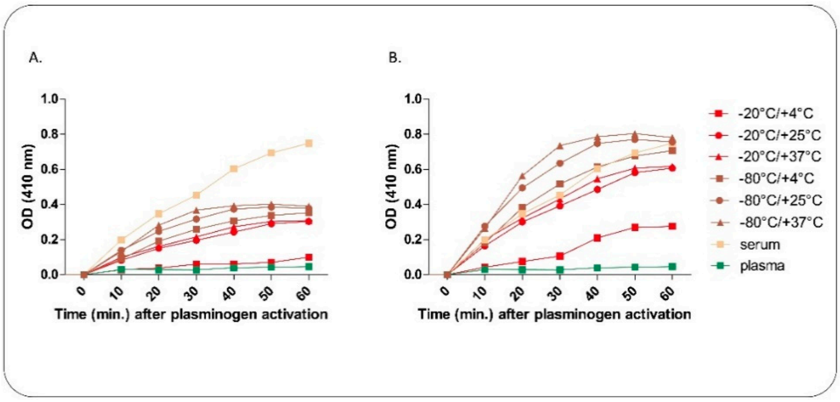

Figure S2. Plasmin activity of frozen GT (A.) and HI (B.) PRF membranes. In case of frozen PRF membrane samples we produced six different samples, with combining the freezing (-20°C or -80°C) and thawing (+4°C, +25°C, +37°C) temperatures.
